# Impact of Insulin Resistance on Retinal Ganglion Cell Layer Structures: Findings from the Korea National Health and Nutrition Examination Survey

**DOI:** 10.3390/jcm15197541

**Published:** 2026-09-28

**Authors:** Young Lee, Je Hyun Seo, Su-Ho Lim

**Affiliations:** 1 Veterans Medical Research Institute, Veterans Health Service Medical Center, Seoul 05368, Republic of Korea; lyou7688@gmail.com; 2 Department of Ophthalmology, Veterans Health Service Medical Center, Seoul 05368, Republic of Korea; 3 Department of Ophthalmology, Daegu Veterans Health Service Medical Center, Daegu 42835, Republic of Korea; mdshlim@gmail.com

**Keywords:** insulin resistance, ganglion cell complex, glaucoma, prediabetes, optical coherence tomography

## Abstract

**Background/Objectives**: Hyperinsulinaemia reflects compensatory systemic insulin resistance and may indicate early metabolic–vascular dysregulation. We investigated associations between insulin dysregulation and the retinal neuronal structure, particularly ganglion cell layer (GCL) thickness, using Korea National Health and Nutrition Examination Survey (KNHANES) data. **Methods**: We analysed KNHANES’s 2019–2020 participants who underwent spectral-domain optical coherence tomography (SD-OCT) and fasting serum insulin assessment. SD-OCT-derived GCL and retinal nerve fibre layer (RNFL) thickness were compared with fasting insulin levels and insulin resistance, which was assessed using the homeostatic model assessment of insulin resistance. Multivariable linear regression models were adjusted for age, sex, body mass index, hypertension, diabetes, and intraocular pressure. To account for multiple comparisons across retinal thickness parameters, a false discovery rate (FDR) correction was applied separately to GCL and RNFL analyses for each exposure and model. **Results**: Among 6941 participants, hyperinsulinaemia was significantly associated with a reduced average GCL thickness after full adjustment (β = −0.106, 95% confidence interval [CI]: −0.179 to −0.032; FDR-adjusted *p* = 0.013). Significant inverse associations remained for seven of the eight GCL parameters after FDR correction, with the superior sector being the only exception. Insulin resistance was associated with a reduced average GCL thickness in the unadjusted model (β = −0.107, 95% CI: −0.169 to −0.045; FDR-adjusted *p* = 0.002), but the association was attenuated after multivariable adjustment and was no longer significant in the fully adjusted model (β = −0.062, 95% CI: −0.125 to 0.001; FDR-adjusted *p* = 0.096). No significant associations with RNFL thickness remained after multivariable adjustment and FDR correction. **Conclusions**: Hyperinsulinaemia was consistently associated with a reduced GCL thickness across most retinal regions, whereas associations between HOMA-IR-defined insulin resistance and GCL thickness were attenuated after multivariable adjustment and FDR correction. No significant associations with RNFL thickness remained after adjustment and multiple-comparison correction. These findings suggest a potential association between hyperinsulinaemia and early GCL thinning, warranting further investigation into its relationship with retinal neurodegeneration and glaucoma risk.

## 1. Introduction

Glaucoma is a leading cause of irreversible blindness worldwide, characterised by progressive retinal ganglion cell loss and corresponding ganglion cell layer (GCL) thinning [1]. In South Korea, its rising prevalence has made glaucoma a major public health concern [2]. Although elevated intraocular pressure (IOP) remains the most important modifiable risk factor [3], increasing evidence suggests that systemic vascular and metabolic factors also contribute to glaucomatous neurodegeneration [4,5].

Insulin resistance, a hallmark of early metabolic dysfunction, is characterised by compensatory hyperinsulinaemia and impaired insulin signalling in peripheral tissues. Beyond glucose metabolism, insulin exerts neurotrophic and neuroprotective effects in the central nervous system, including the retina [6,7]. As a pleiotropic hormone, insulin regulates neuronal survival, synaptic plasticity, and mitochondrial function [8,9]. Clarifying the link between insulin signalling and glaucoma may provide new insight into disease mechanisms and support metabolically targeted preventive strategies. Experimental studies have shown that impaired insulin signalling can promote oxidative stress, chronic inflammation, and apoptosis in neural tissues, all of which are implicated in glaucomatous optic neuropathy [10,11,12].

Several epidemiological studies have reported associations between diabetes mellitus and glaucoma [13,14,15]. Elevated fasting glucose levels have been associated with increased glaucoma risk [16], potentially mediated by microvascular impairment and enhanced oxidative stress in the optic nerve [17] and in trabecular meshwork cells [18]. In the 2005–2008 National Health and Nutrition Examination Survey [15], fasting insulin level showed a dose–response, hockey-stick-shaped association with glaucoma prevalence. This relationship is supported by evidence that insulin resistance, often reflected in the elevated homeostatic model assessment of insulin resistance (HOMA-IR), is linked to greater glaucoma susceptibility. In this regard, one previous study showed that fasting insulin levels and HOMA-IR were associated with IOP [19]. In addition, a recent Mendelian randomisation study showed that higher proinsulin levels were associated with glaucoma risk (odds ratio = 1.263, 95% confidence interval [CI] = 1.020–1.563, *p* = 0.032) [20]. Despite this biological plausibility, few studies have evaluated circulating insulin as a potential blood biomarker for glaucoma. Reliable blood-based biomarkers could provide minimally invasive tools for early detection, risk stratification, and monitoring of disease progression, complementing conventional ophthalmic examinations.

Beyond overt diabetes, subtle alterations in circulating insulin levels and insulin sensitivity may precede clinical disease and reflect early systemic vulnerability to neurodegeneration. The retina shares many structural and functional features with the brain, and insulin receptors are widely expressed in retinal ganglion cells, Müller glia, and retinal pigment epithelium, suggesting that systemic insulin dysregulation may directly affect retinal health. However, the independent effects of insulin resistance, particularly in populations without diabetes, remain insufficiently elucidated [21,22,23]. Therefore, to clarify the association between hyperinsulinaemia and glaucoma, a preliminary evaluation of the relationship between hyperinsulinaemia and retinal structural changes is warranted.

Spectral-domain optical coherence tomography (SD-OCT) enables the quantitative assessment of inner retinal neuronal structures, including the retinal nerve fibre layer (RNFL) and GCL. Moreover, GCL thickness is a sensitive structural marker of early retinal ganglion cell damage. Although previous studies have focused primarily on overt diabetes or glycaemic indices, few population-based studies have examined associations between fasting insulin levels, HOMA-IR, and retinal neuronal thickness. In this context, we investigated associations between insulin resistance and the retinal neuronal structure, focusing on GCL thickness and using Korea National Health and Nutrition Examination Survey (KNHANES) data. We hypothesised that elevated serum insulin levels and HOMA-IR would be independently associated with GCL thinning after adjustment for systemic and ocular risk factors.

## 2. Materials and Methods

### 2.1. Study Design and Participants

The requirement for informed consent was waived by the Institutional Review Board (IRB) of the Veterans Health Service Medical Center (No. 2025-09-014, approval date: 2 October 2025). This study was conducted in accordance with the Declaration of Helsinki.

This retrospective cross-sectional study utilised data from the eighth KNHANES dataset, 2019–2020 (Figure 1). Although the eighth KNHANES was conducted from 2019 to 2021, the 2021 data were excluded because the coronavirus disease pandemic in South Korea limited complete data collection. Among the initially recruited 15,469 participants, those who did not undergo an ophthalmic examination (*n* = 6861) were excluded. The ophthalmic examination relevant to this study was conducted among participants aged 40 years or older; therefore, no additional age-based exclusion was applied. Participants were further excluded for age-related macular degeneration (*n* = 1441), proliferative diabetic retinopathy (*n* = 17), macular oedema (*n* = 13), and central retinal vein occlusion (*n* = 43); and missing insulin data (*n* = 153) (Figure 1). Finally, 6941 participants were included—2937 male participants and 4004 female participants. Of these, 1209 and 2442 participants were classified as having hyperinsulinaemia and insulin resistance, respectively, according to the following criteria.

### 2.2. Measurement of Insulin Levels and Insulin Resistance

Blood samples obtained after a minimum 8 h fast were analysed to measure fasting glucose levels using a Labospect008AS analyser (Hitachi, Tokyo, Japan) and fasting insulin levels using a Cobas 8000 e801 analyser (Roche, Basel, Switzerland). Insulin resistance was assessed using HOMA-IR, calculated as follows: HOMA-IR = fasting insulin (μIU/mL) × fasting glucose (mg/dL)/405. Insulin levels and HOMA-IR were used as the primary exposure variables. Hyperinsulinaemia was defined as values ≥ 13 μIU/mL, and insulin resistance was defined as HOMA-IR ≥ 2.34 [19,24].

### 2.3. Ocular Examination

The Korean Ophthalmological Society (KOS) and the Korea Disease Control and Prevention Agency organised biannual training sessions to ensure the quality of ophthalmic examinations and surveys. Examination quality was validated by the Epidemiology Survey Committee of KOS. During the survey period, trained nurses completed ophthalmic questionnaires and performed examinations. The questionnaires collected data on ophthalmic disease history, ophthalmic surgery history, and current ophthalmic medication use. Ophthalmic examinations included best-corrected visual acuity, IOP, visual field testing (Humphrey frequency doubling technology [FDT] Perimeter; Carl Zeiss Meditec, Dublin, CA, USA), and optical coherence tomography (OCT) (CIRRUS high-definition [HD] OCT 500, Carl Zeiss Meditec). Visual field testing was performed in all participants using FDT according to standard procedures. IOP was measured with participants seated, using Icare PRO (Icare Finland Oy, Helsinki, Finland). For IOP measurement with Icare PRO, participants were instructed to fixate straight ahead while the probe was positioned perpendicular to and 3–7 mm from the corneal centre; six measurements were recorded, and the average was calculated automatically. Experienced specialists selected by the Korean Glaucoma Society and Korean Retinal Society interpreted the ophthalmic examinations and assessed all glaucoma-related and retinal conditions.

For OCT data, posterior segment imaging used Macular Cube 512 × 128 images, two HD 5 Line Raster scans, and the Optic Disc Cube 200 × 200 protocol. The Optic Disc Cube 200 × 200 comprised a 6 × 6 × 2 mm cube centred on the fovea and consisted of 40,000 A-scans. From these scans, OCT parameters, including GCL thickness, RNFL thickness, and macular thickness, were evaluated. The ganglion cell analysis algorithm reported the macular ganglion cell/inner plexiform layer (GCIPL) thickness of six wedge-shaped sectors in a 4.8 × 4.0 mm ellipse, excluding an inner elliptical annulus 1.2 × 1.0 mm in diameter [25]. When the OCT had bad quality, such as signal strength < 6, inaccurate RNFL segmentation or poor disc margin delineation, the OCT was not used as the reference [25]. The variable labelled ‘GC’ in the KNHANES dataset was measured using the Cirrus HD-OCT Ganglion Cell Analysis algorithm, which segments from the outer RNFL boundary to the outer IPL boundary and therefore reflects combined GCIPL thickness rather than an isolated ganglion cell layer [26]. To avoid confusion, we have defined this parameter as ‘GCL’ throughout the present study.

### 2.4. Covariates

The following indices that are potentially associated with both insulin dysregulation and glaucoma were considered as covariates: age, sex, body mass index (BMI), diabetes status, systemic hypertension status, and IOP.

### 2.5. Statistical Analyses

Statistical analyses accounted for the complex KNHANES sampling design, including sample weights, stratification, and clustering. Continuous variables are presented as weighted means with standard errors (SEs), and categorical variables are presented as weighted percentages with SEs. Baseline characteristics were compared by hyperinsulinaemia and insulin resistance status. Continuous variables were compared using design-based *t*-tests, and categorical variables were compared using Rao–Scott chi-square tests.

To examine associations of hyperinsulinaemia and insulin resistance with IOP, linear regression analyses used IOP as the outcome variable. IOP was assessed using two measures—IOP max, defined as the higher value between the right and left eyes, and IOP mean, calculated as the average of both eyes. Sequential models were specified as follows: unadjusted; adjusted for age, sex, and BMI (model 1); and additionally adjusted for diabetes and systemic hypertension status (model 2).

GCL thickness and RNFL thickness were measured in both eyes. For the primary analyses, the mean thickness of the right and left eyes was used for each GCL and RNFL parameter as the participant-level measure. Original GCL and RNFL thickness values were used for descriptive presentation. Before transformation, regression models using the original thickness measurements were examined, and the residual distributions of several GCL and RNFL parameters showed departures from symmetry. Rank-based inverse-normal transformation was therefore applied to the GCL and RNFL measurements for the primary regression analyses; residual histograms after transformation showed more symmetric distributions that were closer to normality. To examine associations of hyperinsulinaemia and insulin resistance with GCL and RNFL thickness, linear regression analyses were performed using sequential models—unadjusted; adjusted for age, sex, and BMI (model 1); additionally adjusted for diabetes and systemic hypertension status (model 2); and further adjusted for IOP values from both eyes (model 3). To account for multiple comparisons across retinal structural parameters, the Benjamini–Hochberg false discovery rate (FDR) procedure was applied separately for hyperinsulinaemia and insulin resistance within each retinal layer and regression model. Specifically, the eight GCL parameters were treated as one testing family, and FDR correction was performed separately for each exposure and each regression model. RNFL parameters were treated as a separate testing family and corrected in the same manner. For GCL and RNFL analyses, statistical significance was determined based on an FDR-adjusted *p*-value < 0.05.

Several additional analyses were performed to assess the robustness and clinical interpretability of the findings. First, sensitivity analyses were performed after excluding participants with glaucoma from the original analytic population. In addition, regression analyses using the untransformed mean GCL thickness values averaged across both eyes were performed in the original analytic population to provide effect estimates on the original micrometre scale. The results of these additional analyses are presented in the Supplementary Materials. All statistical analyses were performed using R version 3.6.3 (R Foundation for Statistical Computing, Vienna, Austria). All tests were two-sided. For baseline comparisons and IOP analyses, a *p* value < 0.05 was considered statistically significant. For GCL and RNFL analyses, statistical significance was determined using an FDR-adjusted *p* value < 0.05.

## 3. Results

### 3.1. Characteristics of the Study Participants

The analysis included 6941 participants with a mean age of 56.30 ± 0.24 years. Insulin dysregulation traits, including hyperinsulinaemia and insulin resistance, and the demographic variables are summarised in Table 1. BMI was significantly higher among participants with hyperinsulinaemia than among controls (27.25 ± 0.12 vs. 23.65 ± 0.05, *p* < 0.001), as well as among participants with insulin resistance than among controls (26.40 ± 0.09 vs. 23.15 ± 0.05, *p* < 0.001). IOP max, defined as the higher value between eyes, was significantly higher among participants with hyperinsulinaemia than among controls (15.35 ± 0.09 vs. 15.06 ± 0.06, *p* = 0.003) and among participants with insulin resistance than among controls (15.35 ± 0.07 vs. 14.99 ± 0.06, *p* < 0.001). A similar pattern was observed for IOP mean (14.88 ± 0.09 vs. 14.56 ± 0.05, *p* < 0.001 for the hyperinsulinaemia comparison; 14.85 ± 0.06 vs. 14.49 ± 0.06, *p* < 0.001 for the insulin resistance comparison).

### 3.2. Associations of Hyperinsulinaemia and Insulin Resistance with IOP

Hyperinsulinaemia was significantly associated with both IOP max (β = 0.288, 95% CI: 0.099 to 0.478; *p* = 0.003) and IOP mean (β = 0.318, 95% CI: 0.140 to 0.496; *p* < 0.001) in the unadjusted regression analysis (Table 2). However, these associations were no longer significant after covariate adjustment in models 1 and 2 (*p* > 0.05). In contrast, insulin resistance was significantly associated with both IOP max (β = 0.356, 95% CI: 0.215 to 0.496; *p* < 0.001) and IOP mean (β = 0.365, 95% CI: 0.232 to 0.499; *p* < 0.001) in the unadjusted regression analysis. These associations remained significant across all adjusted models (ps ≤ 0.001).

### 3.3. Associations of Hyperinsulinaemia with GCL and RNFL Thickness

A comparison of GCL thickness between participants with hyperinsulinaemia and controls showed significantly lower values across all sectors, including the average, minimum, temporal-superior, superior, nasal-superior, nasal-inferior, inferior, and temporal-inferior sectors, in participants with hyperinsulinaemia (Figure 2). However, RNFL thickness did not differ significantly between groups. In regression analyses, hyperinsulinaemia was significantly associated with reduced average GCL thickness in the unadjusted model (β = −0.128, 95% CI: −0.202 to −0.053; FDR-p = 0.003), and this association remained significant after full adjustment (β = −0.106, 95% CI: −0.179 to −0.032; FDR-p = 0.013; Table 3). After full adjustment, significant inverse associations were also observed for the minimum, temporal-superior, nasal-superior, nasal-inferior, inferior, and temporal-inferior GCL thickness (FDR-p < 0.05), whereas the association with superior GCL thickness showed marginal significance (FDR-p = 0.065). In contrast, hyperinsulinaemia was not significantly associated with any RNFL thickness parameter after FDR correction across the regression models (Supplementary Table S1).

### 3.4. Associations of Insulin Resistance with GCL and RNFL Thickness

Compared with controls, participants with insulin resistance showed significantly lower GCL thickness across all eight GCL parameters after FDR correction (Figure 3). Significant differences in RNFL thickness were also observed in most parameters, except the temporal-superior and temporal-inferior sectors. In regression analyses, insulin resistance was significantly associated with a reduced average GCL thickness in the unadjusted model (β = −0.107, 95% CI: −0.169 to −0.045; FDR-p = 0.002; Table 4). Consistent inverse associations were observed for the other seven GCL parameters, with all eight GCL parameters reaching statistical significance after FDR correction in the unadjusted model. In model 1, the associations remained significant for all GCL parameters except the superior sector. However, none of the GCL associations remained statistically significant after FDR correction in models 2 and 3.

For RNFL thickness, significant associations were observed in the unadjusted model for average, minimum, superior, nasal-superior, nasal-inferior, and inferior RNFL thickness after FDR correction, whereas the temporal-superior and temporal-inferior sectors were not significant (Supplementary Table S2). None of the RNFL parameters remained significantly associated with insulin resistance after FDR correction in models 1–3.

### 3.5. Sensitivity Analysis for Associations of Hyperinsulinaemia and Insulin Resistance with Thickness of GCL or RNFL

There was no significant difference in the prevalence of glaucoma (ps > 0.05) or the use of anti-glaucoma eye drops (ps > 0.05) in the hyperinsulinaemia/insulin resistance groups compared with the control group (Table 1). However, since glaucoma itself may affect GCL and RNFL thickness, an additional sensitivity analysis excluding patients with glaucoma was performed to ensure the robustness of the results. Of the initial 6941 participants, 373 with glaucoma were excluded, and the remaining participants were included in the analysis. In regression analyses, hyperinsulinaemia was significantly associated with reduced average GCL thickness in the unadjusted model (β = −0.121, 95% CI: −0.197 to −0.045; FDR-p = 0.007), and this association remained significant after full adjustment (β = −0.108, 95% CI: −0.185 to −0.031; FDR-p = 0.019; Table S3). After full adjustment, significant inverse associations were also observed for the minimum, temporal-superior, nasal-superior, nasal-inferior, and temporal-inferior GCL thickness (FDR-p < 0.05), whereas the association with superior and inferior GCL thickness showed marginal significance (FDR-p = 0.066, 0.084, respectively). In addition, insulin resistance was significantly associated with a reduced average GCL thickness in the unadjusted model and in model 1. However, none of the GCL associations remained statistically significant after FDR correction in models 2 and 3. No RNFL parameters showed a significant association with hyperinsulinaemia (Table S5) or insulin resistance (Table S6) after FDR correction.

### 3.6. Associations of Hyperinsulinaemia and Insulin Resistance with Untransformed GCL Thickness

Given the large sample size of this study, substantial statistical power was achieved, and some statistically significant associations may reflect relatively small differences in magnitude; the clinical relevance of such small differences should therefore be interpreted with caution. To support the robustness of our findings, in addition to the primary analyses using rank-based inverse normal transformed GCL thickness, we performed the same analyses using untransformed GCL thickness values (Tables S7 and S8), which showed results consistent with those obtained using the transformed variable.

## 4. Discussion

In this large, population-based cross-sectional study using KNHANES 2019–2020 data, the associations of two insulin dysregulation phenotypes, hyperinsulinaemia and insulin resistance (HOMA-IR), together with IOP and OCT-measured retinal structural parameters, were investigated. Insulin resistance was independently associated with higher IOP across all adjustment models, whereas the association with hyperinsulinaemia was attenuated after covariate adjustment. Hyperinsulinaemia was independently associated with reduced GCL thickness across most macular sectors after multivariable adjustment with FDR correction, whereas the associations between insulin resistance and GCL thickness were attenuated and no longer statistically significant in the more fully adjusted models. These findings suggest that hyperinsulinaemia may be more consistently associated with GCL structural changes than HOMA-IR-defined insulin resistance. Although, several associations were statistically significant, the absolute differences were relatively modest. Given the large sample size of this population-based study, statistical significance should not necessarily be interpreted as indicating clinical importance.

The independent association between insulin resistance and higher IOP observed in this study is consistent with previous epidemiological evidence. The Hisayama Study, a large, population-based Japanese cohort, showed that HOMA-IR independently predicted higher IOP after adjustment for confounding risk factors [27]. Similarly, a Korean cross-sectional study using KNHANES 2010 data reported a significant positive association between HOMA-IR and IOP in individuals without obesity [28]. An earlier hospital-based study also showed significant positive associations between HOMA scores and IOP after multivariable adjustment [29]. The biological mechanism underlying this association may involve insulin-mediated modulation of aqueous humour dynamics. Insulin resistance is associated with compensatory hyperinsulinaemia and sympathetic nervous system activation, which may promote sodium and fluid retention and thereby increase ciliary body secretion of aqueous humour. Furthermore, insulin resistance-associated endothelial dysfunction and impaired nitric oxide bioavailability in the trabecular meshwork may reduce conventional outflow facility, thereby contributing to IOP elevation.

In contrast to insulin resistance, hyperinsulinaemia was associated with IOP only in the unadjusted model, and this association was not maintained after covariate adjustment, including BMI. This attenuation likely reflects confounding by adiposity, as hyperinsulinaemia and obesity are closely interrelated, and the Hisayama Study similarly reported that obesity status modified the IOP–insulin relationship [27]. The bidirectional relationship between BMI and insulin levels is well documented, with BMI more strongly predicting subsequent insulin levels than vice versa. Therefore, hyperinsulinaemia itself, when separated from its metabolic co-determinants, may not exert an independent effect on IOP regulation strong enough to remain statistically detectable after full adjustment. Importantly, this finding does not exclude a biological role for hyperinsulinaemia in IOP regulation; instead, insulin resistance, as a more integrated marker of metabolic dysfunction, may capture the IOP-relevant metabolic signal more robustly.

Although fasting insulin offers a simple, indirect gauge of insulin resistance, its precision declines in populations with impaired glucose tolerance, a group in which insulin resistance is typically more pronounced. The thresholds used to define hyperinsulinaemia (fasting insulin ≥ 13 μIU/mL) and insulin resistance (HOMA-IR ≥ 2.34) in this study were adopted from Lee et al. [24], who derived reference cutoff values for surrogate measures of insulin resistance in a cohort of Korean non-diabetic adults. In their study, hyperinsulinaemia was defined using the 75th percentile value of the fasting insulin distribution (12.94 μIU/mL), reflecting a population-based distribution-derived threshold, whereas the HOMA-IR cutoff (2.34) was determined by receiver operating characteristic (ROC) curve analysis with the Youden index, representing the value that best discriminated individuals with metabolic syndrome. Although these two thresholds were derived using different statistical approaches, both have been widely adopted in subsequent Korean population-based studies, including several using KNHANES data, and were therefore chosen in the present study to facilitate comparability with the prior literature rather than derive population-specific cutoffs de novo. Nevertheless, we acknowledge that the appropriateness of these thresholds for the present KNHANES cohort warrants discussion. The cutoff values were derived from a relatively selected, non-diabetic health check-up population, which may differ from the nationally representative, community-based KNHANES sample in age distribution, sex ratio, and background metabolic risk profile. Subsequent Korean studies using KNHANES data have reported somewhat different HOMA-IR cutoffs depending on the outcome predicted and the analytic method used—for example, sex- and menopausal status-specific cutoffs ranging from 2.23 to 3.28 for metabolic syndrome [30], and a cutoff of 2.54 for incident type 2 diabetes over a 10-year follow-up [31].

A key finding of this study is that hyperinsulinaemia was independently associated with reduced GCL thickness across most macular sectors after full adjustment with FDR correction, despite showing no independent association with IOP. This dissociation is clinically relevant, as the association between hyperinsulinaemia and GCL thickness persisted after adjustment for IOP, and the underlying mechanisms warrant further investigation. The more consistent association observed for GCL than for RNFL is of interest. One possible explanation is that RGC somata and proximal dendrites may be more vulnerable to early metabolic injury than axons and dependent on insulin/IGF-1–mediated PI3K/Akt survival signalling, whereas the peripapillary RNFL comprises axonal bundles pooled from the entire retina, including peripheral fibres that may be relatively more resistant to early or mild metabolic perturbation; however, this interpretation is biologically plausible but cannot be established from the present cross-sectional data and should therefore be regarded as hypothesis-generating rather than a mechanistic conclusion. Alternative explanations, including differences in measurement characteristics, segmentation algorithms, anatomical variability, and the statistical properties of the two OCT parameters, may also contribute to the observed discordance between GCL and RNFL findings. Finally, this discrepancy is consistent with the broader literature on diabetes-related retinal neurodegeneration. Previous OCT studies of patients with diabetes but no retinopathy have consistently reported thinning of the GCIPL complex, interpreted as early retinal neurodegeneration preceding vascular change [32]. The finding that hyperinsulinaemia is already associated with GCL thinning in the general population extends this concept further upstream. Mechanistically, insulin resistance and hyperinsulinaemia may impair GLUT4-mediated glucose uptake in retinal ganglion cells (RGCs) [33,34], metabolically active neurons that depend on continuous insulin-facilitated glucose supply for survival. Through the PI3K/Akt pathway, insulin also provides antiapoptotic signalling; under insulin-resistant conditions, disruption of this pathway may increase RGC susceptibility to oxidative stress-induced apoptosis. Consistent with this mechanism, diabetes animal models indicate that oxidative stress and cellular senescence can independently drive RGC neurodegeneration [35]. More broadly, diabetic retinal neurodegeneration, mediated through advanced glycation end-product accumulation, glutamate excitotoxicity, and inflammation, may precede vascular diabetic retinopathy, as has previously been reported [36].

Neither hyperinsulinaemia nor insulin resistance showed consistent significant associations with RNFL thickness after multivariable adjustment and FDR correction. This contrasted with the robust GCL associations observed for hyperinsulinaemia, whereas the corresponding associations for insulin resistance were attenuated in the more fully adjusted models. This discordance between GCL and RNFL findings is noteworthy. RGC somata and proximal dendrites may be more vulnerable to early metabolic injury than axonal projections, owing to high somatic metabolic demand and the relative structural resilience of axonal compartments in early disease. Previous studies examining RNFL in metabolic syndrome have reported conflicting results; some found RNFL thinning independently associated with the number of metabolic syndrome components [37] or preclinical diabetes [38], whereas others reported no significant RNFL difference in early diabetes without retinopathy [39]. The absence of a significant association with RNFL thickness warrants further investigation. Future longitudinal studies incorporating repeated OCT measurements are needed to clarify this discrepancy and to determine whether RNFL changes emerge at a later stage of retinal neurodegeneration. Nonetheless, given that this is an observational study that excluded diabetic patients with diabetic retinal complications, this interpretation warrants cautious consideration in light of the aforementioned limitations.

This study has several strengths, including a large, nationally representative sample from KNHANES 2019–2020, standardised OCT measurements, and simultaneous assessment of two insulin dysregulation phenotypes with multiple retinal parameters. Several lines of evidence provide biological context for the observed association between hyperinsulinaemia and GCL thinning. First, retinal neurodegeneration has been reported even in euglycemic hyperinsulinaemia and prediabetes, prior to overt diabetes or retinopathy, suggesting a direct effect of insulin dysregulation independent of hyperglycaemia [40]. Second, mechanistic studies implicate oxidative stress, chronic low-grade inflammation, endothelial dysfunction, and impaired insulin/IGF-1-mediated trophic signalling as pathways linking insulin resistance to ganglion cell injury [11,41], consistent with the sympathetic activation and reduced nitric oxide bioavailability discussed above. Third, independent NHANES-based studies using the triglyceride-glucose index, a different insulin resistance surrogate, have similarly reported associations with glaucoma risk [16], supporting its generalizability across populations and metrics. Nevertheless, some limitations merit discussion. First, the cross-sectional design precludes causal inference and does not establish whether insulin dysregulation precedes GCL loss; we cannot determine whether insulin dysregulation precedes GC layer thinning or whether an alternative temporal or bidirectional relationship exists. Longitudinal studies are needed to clarify the temporal relationship between insulin dysregulation and retinal neurodegeneration, and to determine whether hyperinsulinaemia or insulin resistance predicts subsequent structural changes. In addition, the large sample size may have allowed detection of relatively small differences that are statistically significant but of uncertain clinical relevance. Second, fasting serum insulin levels, although validated as a surrogate for hyperinsulinaemia, may not fully capture postprandial insulin dynamics. Third, participants with known proliferative diabetic retinopathy were excluded, but subclinical glycaemic abnormalities could not be fully excluded. Fourth, OCT measurements were obtained in a single visit, and intra-session variability was not assessed. Clinically, our findings suggest that hyperinsulinaemia, may be associated with GCL thinning that mirrors patterns observed in early glaucomatous or diabetic neurodegeneration. However, GCL thinning does not necessarily equate to a diagnosis of glaucoma, as structural change may precede clinically detectable glaucomatous damage by a considerable interval; thus, although glaucoma diagnosis and visual field data were available in KNHANES, GCL thinning alone cannot be used to immediately determine glaucoma status, and should instead be interpreted as a potential early structural marker warranting further evaluation. Longitudinal studies incorporating OCT/OCT angiography, visual field testing, IOP, metabolic biomarkers, and standardised glaucoma assessment are required to determine whether hyperinsulinaemia-associated GC layer thinning predicts subsequent glaucomatous structural and functional progression.

## 5. Conclusions

Hyperinsulinaemia, reflected by elevated fasting insulin, was associated with GCL thinning but not RNFL thinning in a large, nationally representative population. Associations between HOMA-IR-defined insulin resistance and GCL thickness were attenuated after multivariable adjustment with FDR correction. These findings suggest that a potential association between hyperinsulinaemia and early GCL thinning, which may warrant further investigation into the relationship between metabolic dysfunction and GCL thickness. However, given the cross-sectional design of this study, causal inference cannot be made, and longitudinal studies are needed to determine whether insulin dysregulation predicts subsequent retinal neuronal loss or the development of glaucoma.

## Figures and Tables

**Figure 1:**
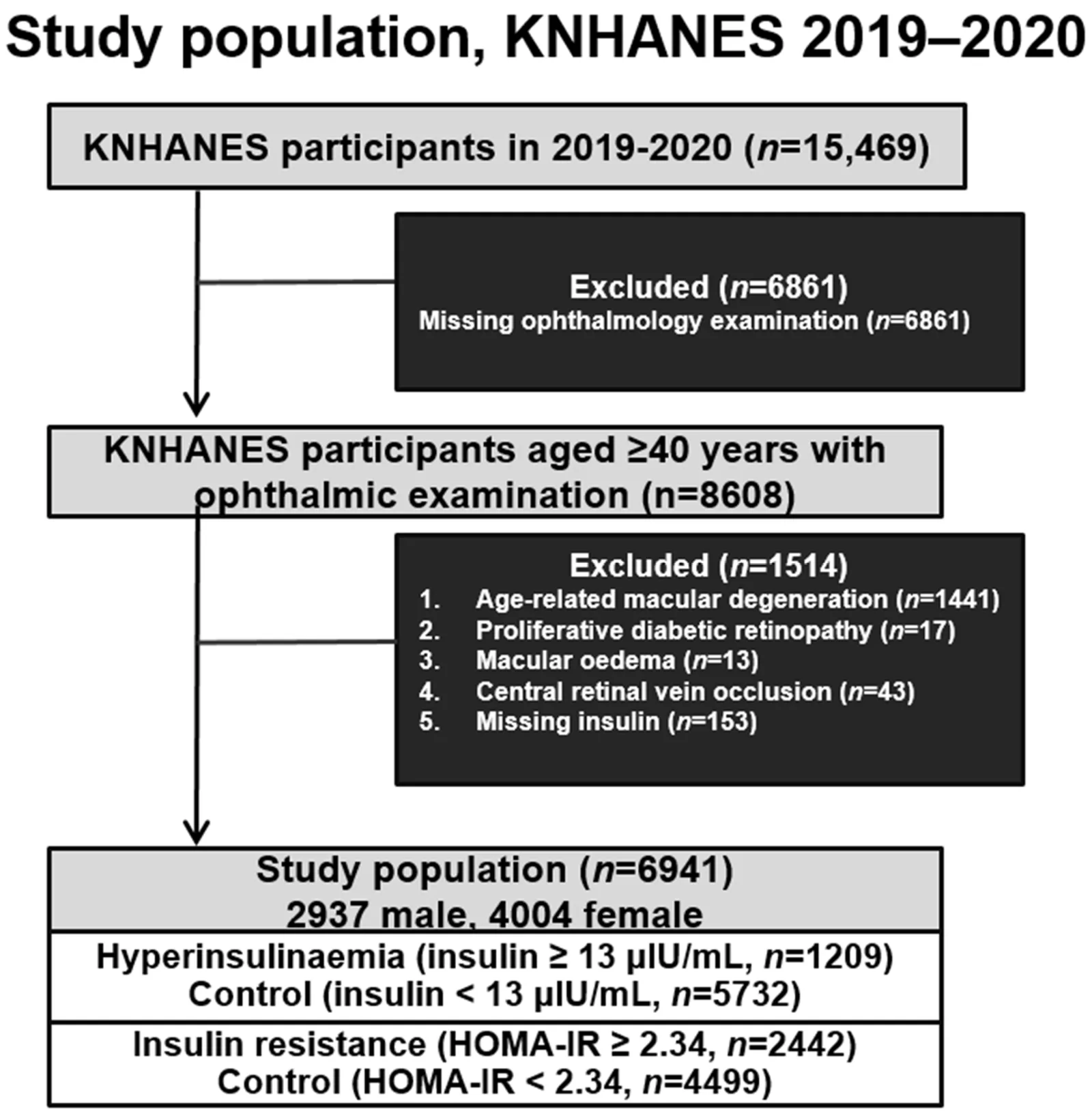
Flowchart of the study population. KNHANES, Korea National Health and Nutrition Examination Survey; HOMA-IR, homeostatic model assessment of insulin resistance.

**Figure 2:**
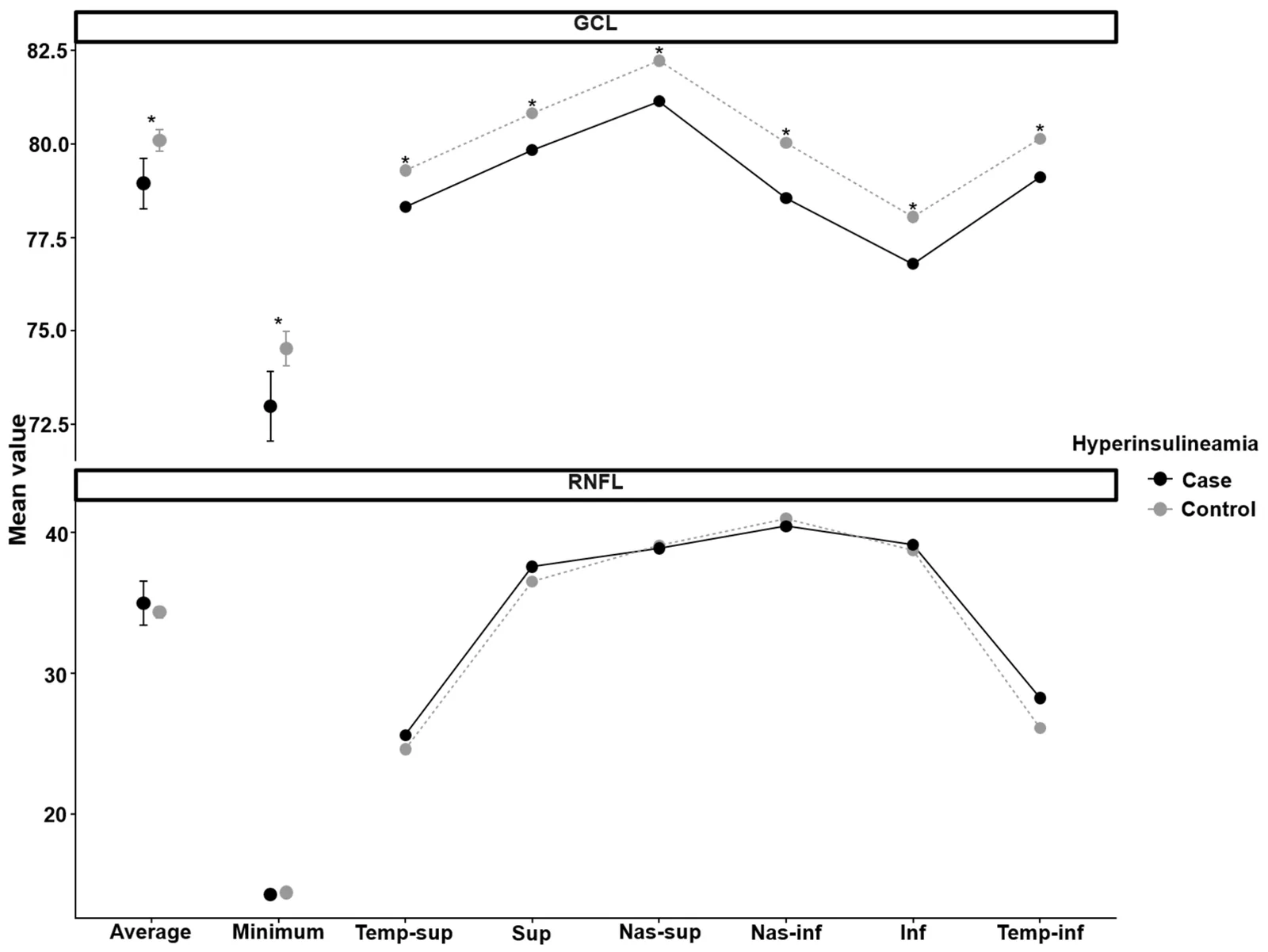
GCL thickness and RNFL thickness differences according to the hyperinsulinaemia status. Data points indicate mean values of the original GCL and RNFL thickness measurements. Error bars for average and minimum thickness indicate 95% confidence intervals. Asterisks (*) indicate statistically significant differences between the hyperinsulinaemia and control groups based on analyses of rank-based inverse normal transformed variables, with significance determined using an FDR-p value < 0.05. For each GCL and RNFL thickness parameter, the mean value of the right and left eyes was used in the analysis. GCL, ganglion cell layer; RNFL, retinal nerve fibre layer; Sup, superior; Inf, inferior; Temp, temporal. Hyperinsulinaemia was defined as values ≥ 13 μIU/mL.

**Figure 3:**
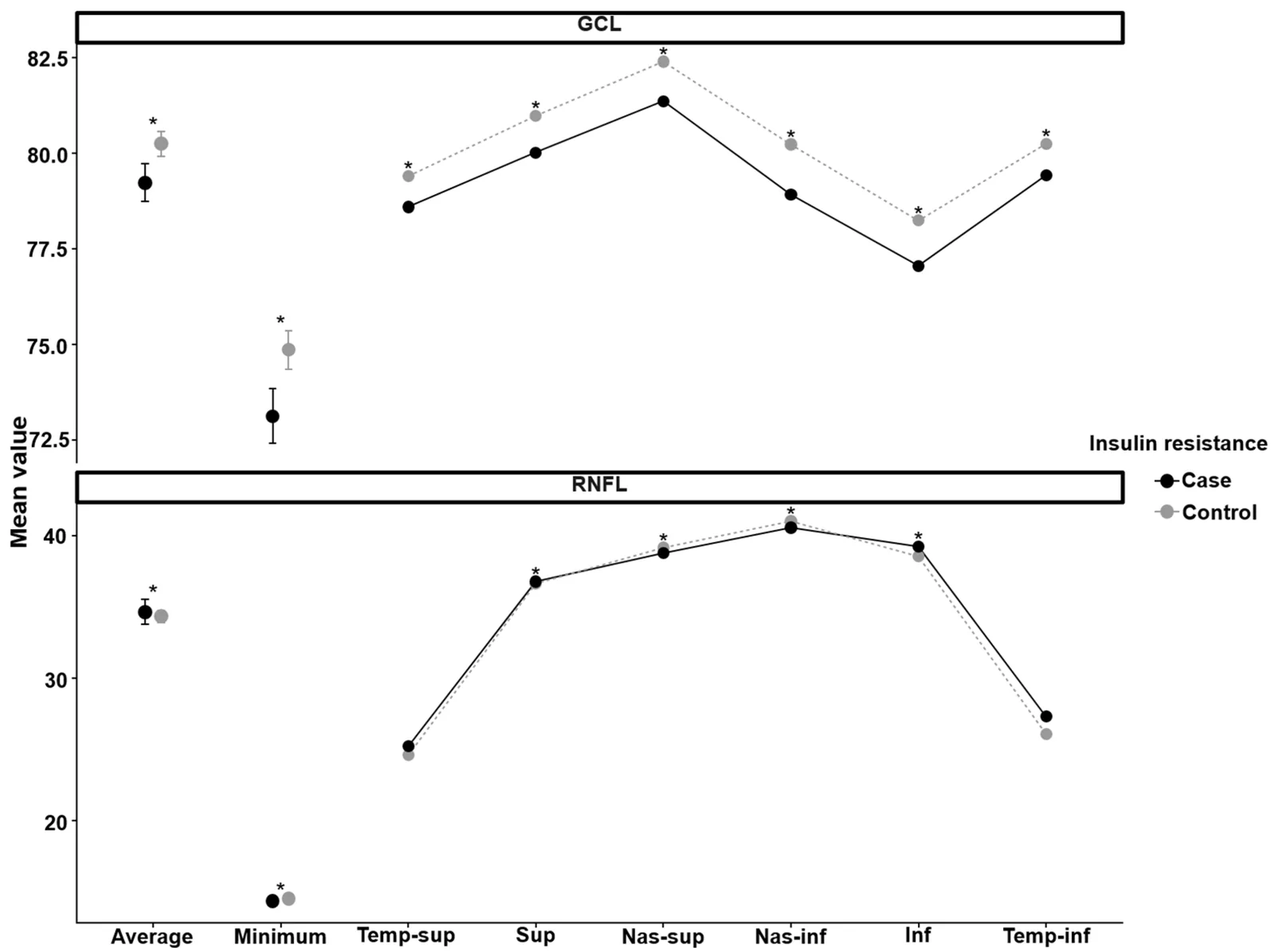
GCL thickness and RNFL thickness differences according to the insulin resistance status. Data points indicate mean values of the original GCL and RNFL thickness measurements. Error bars for average and minimum thickness indicate 95% confidence intervals. Asterisks (*) indicate statistically significant differences between the insulin resistance and control groups based on analyses of rank-based inverse normal transformed variables, with significance determined using an FDR-p value < 0.05. For each GCL and RNFL thickness parameter, the mean value of the right and left eyes was used in the analysis. GCL, ganglion cell layer; RNFL, retinal nerve fibre layer; Sup, superior; Inf, inferior; Temp, temporal. Insulin resistance was defined as HOMA-IR ≥ 2.34.

**Table 1:** Demographics of the study participants according to hyperinsulinaemia and insulin resistance.

Variable	Total (*n* = 6941)	Hyperinsulinaemia Comparison			Insulin Resistance Comparison		
		Hyperinsulinaemia (*n* = 1209)	Control (*n* = 5732)	*p*	Insulin resistance (*n* = 2442)	Control (*n* = 4499)	*p*
Age	56.30 ± 0.24	56.85 ± 0.37	56.18 ± 0.26	0.079	57.22 ± 0.31	55.80 ± 0.26	<0.001
Sex % (male)	47.69 (0.552)	51.91 (1.645)	46.80 (0.627)	0.006	53.93 (1.084)	44.35 (0.731)	<0.001
Body mass index	24.28 ± 0.05	27.25 ± 0.12	23.65 ± 0.05	<0.001	26.40 ± 0.09	23.15 ± 0.05	<0.001
Drink (non-drinker)	29.82 (0.727)	32.92 (1.698)	29.16 (0.775)	0.034	30.30 (1.167)	29.56 (0.855)	0.584
Smoking (non-smoker, ex-smoker)	81.92 (0.602)	81.26 (1.459)	82.06 (0.624)	0.593	79.87 (1.119)	83.03 (0.663)	0.010
Comorbidity							
Systemic hypertension	28.29 (0.740)	46.91 (1.811)	24.32 (0.775)	<0.001	41.72 (1.319)	21.08 (0.793)	<0.001
Dyslipidaemia	24.70 (0.647)	36.68 (1.564)	22.14 (0.679)	<0.001	33.18 (1.136)	20.14 (0.748)	<0.001
Diabetes	11.88 (0.474)	18.45 (1.339)	10.47 (0.499)	<0.001	19.92 (0.995)	7.56 (0.492)	<0.001
Stroke	2.25 (0.194)	2.85 (0.529)	2.12 (0.219)	0.188	2.75 (0.356)	1.99 (0.242)	0.077
Sleep apnoea	0.62 (0.116)	0.82 (0.289)	0.58 (0.117)	0.360	0.73 (0.190)	0.56 (0.133)	0.433
Snoring	20.37 (0.610)	32.5 (1.711)	17.83 (0.628)	<0.001	28.88 (1.234)	15.88 (0.646)	<0.001
Glaucoma prevalence	5.13 (0.337)	5.54 (0.734)	5.04 (0.371)	0.528	5.55 (0.535)	4.90 (0.411)	0.319
Number of glaucoma medications	1.71 (0.211)	2.04 (0.611)	1.63 (0.213)	0.488	2.16 (0.406)	1.46 (0.234)	0.105
Laboratory finding							
Glucose level	104.30 ± 0.40	115.5 ± 1.22	101.91 ± 0.37	<0.001	117.60 ± 0.90	97.17 ± 0.27	<0.001
Insulin level	9.13 ± 0.12	20.74 ± 0.43	6.65 ± 0.05	<0.001	15.61 ± 0.25	5.64 ± 0.04	<0.001
HOMA-IR	2.47 ± 0.04	6.07 ± 0.16	1.70 ± 0.01	<0.001	4.55 ± 0.09	1.35 ± 0.01	<0.001
Total cholesterol	195.15 ± 0.57	190.63 ± 1.44	196.12 ± 0.62	<0.001	192.91 ± 0.96	196.36 ± 0.68	0.003
TG	143.43 ± 1.77	192.24 ± 5.74	133.02 ± 1.72	<0.001	182.83 ± 3.35	122.29 ± 1.80	<0.001
LDL	114.48 ± 1.30	113.77 ± 2.42	114.83 ± 1.58	0.718	114.57 ± 1.59	114.34 ± 2.17	0.932
HDL	51.36 ± 0.21	45.54 ± 0.35	52.60 ± 0.22	<0.001	46.71 ± 0.26	53.85 ± 0.24	<0.001
Ocular examination							
IOP max	15.11 ± 0.05	15.35 ± 0.09	15.06 ± 0.06	0.003	15.35 ± 0.07	14.99 ± 0.06	<0.001
IOP mean	14.61 ± 0.05	14.88 ± 0.09	14.56 ± 0.05	<0.001	14.85 ± 0.06	14.49 ± 0.06	<0.001
AXL Rt	24.01 ± 0.03	24.06 ± 0.05	24.00 ± 0.03	0.269	24.03 ± 0.04	24.00 ±0.03	0.559
AXL Lt	24.01 ± 0.03	24.07 ± 0.05	24.00 ± 0.03	0.225	24.02 ± 0.04	24.01 ± 0.04	0.761
CCT Rt	550.42 ± 0.71	551.80 ± 1.53	550.13 ± 0.74	0.295	552.06 ± 1.07	549.55 ± 0.83	0.045
CCT Lt	549.82 ± 0.65	551.30 ± 1.45	549.51 ± 0.71	0.257	551.55 ± 1.05	548.91 ± 0.78	0.036

AXL, axial length; CCT, central corneal thickness; HOMA-IR, homeostatic model assessment of insulin resistance; TG, triglyceride; LDL, low-density lipoprotein; HDL, high-density lipoprotein; IOP, intraocular pressure.

**Table 2:** Association between hyperinsulinaemia/insulin resistance and IOP.

		Unadjusted		Model 1		Model 2	
		β (95% CI)	*p*	β (95% CI)	*p*	β (95% CI)	*p*
Hyperinsulinaemia	IOP max	0.288 (0.099, 0.478)	0.003	0.135 (−0.066, 0.336)	0.188	0.115 (−0.088, 0.318)	0.267
	IOP mean	0.318 (0.140, 0.496)	<0.001	0.173 (−0.015, 0.362)	0.071	0.156 (−0.033, 0.346)	0.106
Insulin resistance	IOP max	0.356 (0.215, 0.496)	<0.001	0.285 (0.133, 0.438)	<0.001	0.259 (0.102, 0.416)	0.001
	IOP mean	0.365 (0.232, 0.499)	<0.001	0.304 (0.160, 0.449)	<0.001	0.281 (0.134, 0.428)	<0.001

β coefficients represent the difference in IOP compared with the reference group without hyperinsulinaemia or insulin resistance: unadjusted; model 1—adjusted for age, sex, and BMI; model 2— same as model 1 but additionally adjusted for the presence of diabetes and systemic hypertension. CI, confidence interval; HOMA-IR, homeostatic model assessment of insulin resistance. Hyperinsulinaemia was defined as values ≥ 13 μIU/mL and insulin resistance as HOMA-IR ≥ 2.34.

**Table 3:** Association between hyperinsulinaemia and GCL thickness.

	Unadjusted		Model 1		Model 2		Model 3	
	β (95% CI)	FDR-p	β (95% CI)	FDR-p	β (95% CI)	FDR-p	β (95% CI)	FDR-p
GCL Average	−0.128 (−0.202, −0.053)	0.003	−0.126 (−0.199, −0.053)	0.002	−0.110 (−0.183, −0.036)	0.009	−0.106 (−0.179, −0.032)	0.013
GCL Minimum	−0.123 (−0.199, −0.046)	0.005	−0.101 (−0.175, −0.027)	0.010	−0.082 (−0.156, −0.008)	0.037	−0.078 (−0.153, −0.003)	0.049
GCL Temp-Sup	−0.108 (−0.181, −0.035)	0.005	−0.129 (−0.202, −0.056)	0.002	−0.114 (−0.188, −0.041)	0.009	−0.112 (−0.186, −0.037)	0.013
GCL Sup	−0.097 (−0.174, −0.019)	0.014	−0.092 (−0.169, −0.015)	0.019	−0.080 (−0.158, −0.003)	0.043	−0.074 (−0.152, 0.005)	0.065
GCL Nasal-Sup	−0.111 (−0.187, −0.034)	0.005	−0.108 (−0.187, −0.030)	0.010	−0.097 (−0.176, −0.018)	0.027	−0.092 (−0.171, −0.013)	0.035
GCL Nasal-Inf	−0.148 (−0.222, −0.074)	<0.001	−0.128 (−0.203, −0.054)	0.002	−0.112 (−0.187, −0.038)	0.009	−0.109 (−0.184, −0.035)	0.013
GCL Inf	−0.115 (−0.190, −0.041)	0.005	−0.097 (−0.171, −0.023)	0.012	−0.081 (−0.156, −0.007)	0.037	−0.078 (−0.153, −0.002)	0.049
GCL Temp-Inf	−0.106 (−0.179, −0.033)	0.005	−0.118 (−0.193, −0.042)	0.005	−0.101 (−0.177, −0.025)	0.018	−0.096 (−0.172, −0.020)	0.027

For each GC layer thickness parameter, the mean value of the right and left eyes was used in the analysis. GC layer thickness variables were rank-based inverse normal transformed before analysis. β coefficients represent differences in transformed GC layer thickness values compared with participants without hyperinsulinaemia: unadjusted; adjusted for age, sex, and BMI (model 1); model 1 additionally adjusted for the presence of diabetes and systemic hypertension (model 2); model 2 additionally adjusted for IOP (model 3). BMI, body mass index; CI, confidence interval; FDR-p, false discovery rate adjusted *p*; GCL, ganglion cell layer; Sup, superior; Inf, inferior; Temp, temporal. Hyperinsulinaemia was defined as values ≥ 13 μIU/mL.

**Table 4:** Association between insulin resistance and GCL thickness.

	Unadjusted		Model 1		Model 2		Model 3	
	β (95% CI)	FDR-p	β (95% CI)	FDR-p	β (95% CI)	FDR-p	β (95% CI)	FDR-p
GCL Average	−0.107 (−0.169, −0.045)	0.002	−0.082 (−0.145, −0.020)	0.020	−0.061 (−0.124, 0.002)	0.109	−0.062 (−0.125, 0.001)	0.096
GCL Minimum	−0.122 (−0.185, −0.059)	<0.001	−0.086 (−0.148, −0.024)	0.020	−0.058 (−0.121, 0.005)	0.109	−0.059 (−0.122, 0.004)	0.096
GCL Temp-Sup	−0.078 (−0.138, −0.017)	0.013	−0.082 (−0.146, −0.018)	0.020	−0.063 (−0.128, 0.001)	0.109	−0.064 (−0.129, 0.001)	0.096
GCL Sup	−0.090 (−0.152, −0.029)	0.005	−0.062 (−0.124, 0.000)	0.052	−0.043 (−0.105, 0.020)	0.181	−0.041 (−0.104, 0.022)	0.199
GCL Nasal-Sup	−0.097 (−0.157, −0.037)	0.003	−0.071 (−0.134, −0.008)	0.030	−0.055 (−0.118, 0.008)	0.109	−0.054 (−0.117, 0.009)	0.106
GCL Nasal-Inf	−0.127 (−0.188, −0.066)	<0.001	−0.083 (−0.145, −0.021)	0.020	−0.063 (−0.126, 0.001)	0.109	−0.063 (−0.127, 0.000)	0.096
GCL Inf	−0.112 (−0.173, −0.050)	0.001	−0.075 (−0.138, −0.012)	0.026	−0.054 (−0.118, 0.010)	0.109	−0.059 (−0.123, 0.005)	0.096
GCL Temp-Inf	−0.085 (−0.145, −0.025)	0.006	−0.085 (−0.147, −0.022)	0.020	−0.065 (−0.128, −0.002)	0.109	−0.068 (−0.131, −0.005)	0.096

For each GC layer thickness parameter, the mean value of the right and left eyes was used in the analysis. GC layer thickness variables were rank-based inverse normal transformed before analysis. β coefficients represent differences in transformed GC layer thickness values compared with participants without insulin resistance: unadjusted; adjusted for age, sex, and BMI (model 1); model 1 additionally adjusted for presence of diabetes, presence of systemic hypertension (model 2); model 2 additionally adjusted for IOP (model 3). BMI, body mass index; CI, confidence interval; FDR-p, false discovery rate adjusted *p*; HOMA-IR, homeostatic model assessment of insulin resistance; GCL, ganglion cell layer; Sup, superior; Inf, inferior; Temp, temporal. Insulin resistance was defined as HOMA-IR ≥ 2.34.

## Data Availability

The data that support this study are available in the Korea National Health and Nutrition Examination Survey (KNHANES) (https://knhanes.kdca.go.kr/knhanes/eng/main.do, accessed on 3 February 2026). These data were derived from resources available in the public domain.

## Supplementary Materials

The following supporting information can be downloaded at: https://www.mdpi.com/article/10.3390/jcm15197541/s1, Table S1: Association between hyperinsulinaemia and retinal nerve fibre layer thickness; Table S2: Association between insulin resistance and retinal nerve fibre layer thickness; Table S3: Senstivity analysis for association between hyperinsulinaemia and GCL thickness by excluding glaucoma patients; Table S4: Senstivity analysis for association between insulin resistance and GCL thickness by excluding glaucoma patients; Table S5: Senstivity analysis for association between hyperinsulinaemia and retinal nerve fibre layer thickness by excluding glaucoma patients; Table S6: Senstivity analysis for association between insulin resistance and retinal nerve fibre layer thickness by excluding glaucoma patients; Table S7: Association between hyperinsulinaemia and GCL thickness using untransformed regression analyses; Table S8: Association between insulin resistance and GCL thickness using untransformed regression analyses.

## Abbreviations

The following abbreviations are used in this manuscript:

AXL	Axial length
BMI	Body mass index
CCT	Central corneal thickness
CI	Confidence interval
FDT	Frequency doubling technology
FDR	False discovery rate
GCL	Ganglion cell layer
HDL	High-density lipoprotein
HOMA-IR	Homeostatic model assessment of insulin resistance
IOP	Intraocular pressure
KNHANES	Korea National Health and Nutrition Examination Survey
LDL	Low-density lipoprotein
OCT	Optical coherence tomography
RNFL	Retinal nerve fibre layer
SD-OCT	Spectral-domain optical coherence tomography
SE	Standard error
TG	Triglyceride
