# Supplementary material for: Impact of Insulin Resistance on Retinal Ganglion Cell Layer Structures: Findings from the Korea National Health and Nutrition Examination Survey
Source: J Clin Med. 2026 Sep 28;15(19):7541. doi: 10.3390/jcm15197541 (PMC13665767; doi:10.3390/jcm15197541)
Supplement: Supplementary file 1 [file jcm-15-07541-s001.zip › jcm-4516575-supplementary.pdf]

Table S1. Association between hyperinsulinaemia and retinal nerve fibre layer thickness

	Unadjusted		Model 1		Model 2		Model 3	
	β (95% CI)	FDR-p	β (95% CI)	FDR-p	β (95% CI)	FDR-p	β (95% CI)	FDR-p
**RNFL Average**	-0.069 (-0.141, 0.003)	0.122	-0.074 (-0.148, 0.000)	0.078	-0.063 (-0.137, 0.011)	0.153	-0.056 (-0.130, 0.018)	0.202
**RNFL Mininum**	-0.096 (-0.171, -0.021)	0.098	-0.106 (-0.186, -0.026)	0.078	-0.098 (-0.179, -0.018)	0.132	-0.098 (-0.179, -0.017)	0.140
**RNFL Temp-sup**	-0.057 (-0.127, 0.014)	0.179	-0.081 (-0.155, -0.007)	0.078	-0.071 (-0.146, 0.004)	0.150	-0.064 (-0.137, 0.010)	0.202
**RNFL Sup**	-0.047 (-0.117, 0.024)	0.194	-0.033 (-0.105, 0.038)	0.354	-0.019 (-0.091, 0.053)	0.598	-0.015 (-0.088, 0.057)	0.678
**RNFL Nasal-sup**	-0.057 (-0.131, 0.018)	0.179	-0.039 (-0.113, 0.035)	0.345	-0.029 (-0.103, 0.046)	0.513	-0.023 (-0.096, 0.050)	0.609
**RNFL Nasal-inf**	-0.072 (-0.144, 0.000)	0.122	-0.063 (-0.137, 0.011)	0.124	-0.056 (-0.130, 0.017)	0.178	-0.054 (-0.129, 0.020)	0.202
**RNFL Inf**	-0.079 (-0.149, -0.009)	0.107	-0.084 (-0.157, -0.012)	0.078	-0.073 (-0.146, 0.000)	0.150	-0.072 (-0.145, 0.002)	0.202
**RNFL Temp-inf**	-0.052 (-0.127, 0.024)	0.194	-0.085 (-0.166, -0.003)	0.078	-0.074 (-0.155, 0.008)	0.150	-0.067 (-0.149, 0.015)	0.202

For each RNFL thickness parameter, the mean value of the right and left eyes was used in the analysis. RNFL thickness variables were rank-based inverse normal transformed before analysis. β coefficients represent differences in transformed RNFL thickness values compared with participants without hyperinsulinemia.

unadjusted; adjusted for age, sex and BMI (model 1); model 1 additionally adjusted for presence of diabetes, presence of systemic hypertension (model 2); model 2 additionally adjusted for IOP (model 3)

BMI, body mass index; CI, Confidence Interval; FDR-p, false discovery rate adjusted; Sup, superior; Inf, inferior; Temp, temporal; RNFL, retinal nerve fibre layer

Hyperinsulinaemia was defined as values ≥ 13 μIU/mL

Table S2. Association between insulin resistance and retinal nerve fibre layer thickness

	Unadjusted		Model 1		Model 2		Model 3	
	**β (95% CI)**	**FDR-p**	**β (95% CI)**	**FDR-p**	**β (95% CI)**	**FDR-p**	**β (95% CI)**	**FDR-p**
**RNFL Average**	-0.060 (-0.116, -0.004)	0.046	-0.059 (-0.118, 0.000)	0.106	-0.045 (-0.103, 0.014)	0.279	-0.040 (-0.099, 0.019)	0.367
**RNFL Mininum**	-0.075 (-0.135, -0.015)	0.046	-0.078 (-0.144, -0.013)	0.106	-0.063 (-0.128, 0.003)	0.279	-0.064 (-0.131, 0.003)	0.367
**RNFL Temp-sup**	-0.032 (-0.089, 0.025)	0.312	-0.046 (-0.108, 0.016)	0.198	-0.033 (-0.096, 0.030)	0.398	-0.032 (-0.094, 0.030)	0.414
**RNFL Sup**	-0.063 (-0.121, -0.005)	0.046	-0.040 (-0.102, 0.021)	0.227	-0.026 (-0.087, 0.035)	0.461	-0.026 (-0.087, 0.036)	0.473
**RNFL Nasal-sup**	-0.077 (-0.135, -0.019)	0.046	-0.060 (-0.120, 0.000)	0.106	-0.053 (-0.113, 0.008)	0.279	-0.050 (-0.110, 0.010)	0.367
**RNFL Nasal-inf**	-0.063 (-0.120, -0.006)	0.046	-0.044 (-0.103, 0.016)	0.198	-0.035 (-0.095, 0.025)	0.398	-0.034 (-0.094, 0.027)	0.414
**RNFL Inf**	-0.059 (-0.113, -0.006)	0.046	-0.057 (-0.116, 0.001)	0.106	-0.044 (-0.103, 0.014)	0.279	-0.044 (-0.104, 0.015)	0.367
**RNFL Temp-inf**	-0.006 (-0.062, 0.051)	0.845	-0.040 (-0.104, 0.025)	0.227	-0.020 (-0.085, 0.045)	0.538	-0.018 (-0.084, 0.049)	0.603

For each RNFL thickness parameter, the mean value of the right and left eyes was used in the analysis. RNFL thickness variables were rank-based inverse normal transformed before analysis. β coefficients represent differences in transformed RNFL thickness values compared with participants without insulin resistance.

unadjusted; adjusted for age, sex and BMI (model 1); model 1 additionally adjusted for presence of diabetes, presence of systemic hypertension (model 2); model 2 additionally adjusted for IOP (model 3)

BMI, body mass index; CI, Confidence Interval; FDR-p, false discovery rate adjusted; HOMA-IR, homeostatic model assessment of insulin resistance; Sup, superior; Inf, inferior; Temp, temporal; RNFL, retinal nerve fibre layer

Insulin resistance was defined as HOMA-IR ≥ 2.34

Table S3. Senstivity analysis for association between hyperinsulinaemia and GCL thickness by excluding glaucoma patients

	Unadjusted		Model 1		Model 2		Model 3	
	β (95% CI)	FDR-p	β (95% CI)	FDR-p	β (95% CI)	FDR-p	β (95% CI)	FDR-p
**GCL Average**	-0.121 (-0.197, -0.045)	0.007	-0.122 (-0.199, -0.046)	0.007	-0.110 (-0.187, -0.033)	0.017	-0.108 (-0.185, -0.031)	0.019
**GCL Minimum**	-0.120 (-0.198, -0.041)	0.007	-0.105 (-0.182, -0.028)	0.011	-0.090 (-0.168, -0.012)	0.033	-0.087 (-0.166, -0.008)	0.042
**GCL Temp-sup**	-0.103 (-0.177, -0.030)	0.010	-0.130 (-0.204, -0.055)	0.006	-0.119 (-0.195, -0.043)	0.017	-0.117 (-0.194, -0.040)	0.019
**GCL Sup**	-0.090 (-0.169, -0.011)	0.025	-0.091 (-0.170, -0.012)	0.027	-0.083 (-0.164, -0.003)	0.049	-0.078 (-0.159, 0.003)	0.066
**GCL Nasal-sup**	-0.116 (-0.193, -0.038)	0.007	-0.113 (-0.194, -0.033)	0.009	-0.105 (-0.187, -0.024)	0.019	-0.103 (-0.184, -0.022)	0.026
**GCL Nasal-inf**	-0.136 (-0.213, -0.060)	0.004	-0.120 (-0.198, -0.043)	0.007	-0.109 (-0.187, -0.031)	0.017	-0.107 (-0.185, -0.029)	0.019
**GCL Inf**	-0.102 (-0.179, -0.025)	0.011	-0.085 (-0.163, -0.007)	0.033	-0.072 (-0.151, 0.006)	0.072	-0.070 (-0.149, 0.009)	0.084
**GCL Temp-inf**	-0.099 (-0.172, -0.025)	0.011	-0.115 (-0.193, -0.037)	0.008	-0.101 (-0.180, -0.023)	0.019	-0.097 (-0.176, -0.018)	0.026

After excluding 373 participants with glaucoma from the initial cohort of 6,941, the sensitivity analysis was performed on the remaining participants.

For each GC layer thickness parameter, the mean value of the right and left eyes was used in the analysis. GC layer thickness variables were rank-based inverse normal transformed before analysis. β coefficients represent differences in transformed GC layer thickness values compared with participants without hyperinsulinaemia;unadjusted; adjusted for age, sex and BMI (model 1); model 1 additionally adjusted for presence of diabetes, presence of systemic hypertension (model 2); model 2 additionally adjusted for IOP (model 3); BMI, body mass index; CI, Confidence Interval; FDR-p, false discovery rate adjusted p; GCL, Ganglion Cell Layer; Sup, superior; Inf, inferior; Temp, temporal;Hyperinsulinaemia was defined as values ≥ 13 μIU/mL

Table S4. Senstivity analysis for association between insulin resistance and GCL thickness by excluding glaucoma patients

	Unadjusted		Model 1		Model 2		Model 3	
	β (95% CI)	FDR-p	β (95% CI)	FDR-p	β (95% CI)	FDR-p	β (95% CI)	FDR-p
**GCL Average**	-0.101 (-0.165, -0.038)	0.004	-0.080 (-0.145, -0.015)	0.031	-0.064 (-0.129, 0.001)	0.111	-0.106 (-0.179, -0.032)	0.099
**GCL Minimum**	-0.119 (-0.183, -0.055)	0.002	-0.089 (-0.152, -0.026)	0.023	-0.065 (-0.130, -0.001)	0.111	-0.078 (-0.153, -0.003)	0.099
**GCL Temp-sup**	-0.078 (-0.140, -0.016)	0.013	-0.089 (-0.154, -0.025)	0.023	-0.076 (-0.141, -0.011)	0.111	-0.112 (-0.186, -0.037)	0.099
**GCL Sup**	-0.088 (-0.151, -0.025)	0.008	-0.065 (-0.128, -0.001)	0.048	-0.052 (-0.116, 0.012)	0.130	-0.074 (-0.152, 0.005)	0.120
**GCL Nasal-sup**	-0.097 (-0.160, -0.034)	0.004	-0.070 (-0.136, -0.004)	0.048	-0.059 (-0.125, 0.008)	0.130	-0.092 (-0.171, -0.013)	0.120
**GCL Nasal-inf**	-0.112 (-0.176, -0.048)	0.003	-0.069 (-0.133, -0.004)	0.048	-0.054 (-0.119, 0.012)	0.130	-0.109 (-0.184, -0.035)	0.120
**GCL Inf**	-0.101 (-0.165, -0.038)	0.004	-0.065 (-0.130, -0.001)	0.048	-0.049 (-0.114, 0.017)	0.143	-0.078 (-0.153, -0.002)	0.120
**GCL Temp-inf**	-0.080 (-0.140, -0.020)	0.011	-0.085 (-0.148, -0.022)	0.023	-0.070 (-0.134, -0.006)	0.111	-0.096 (-0.172, -0.020)	0.099

After excluding 373 participants with glaucoma from the initial cohort of 6,941, the sensitivity analysis was performed on the remaining participants.

For each GC layer thickness parameter, the mean value of the right and left eyes was used in the analysis. GC layer thickness variables were rank-based inverse normal transformed before analysis. β coefficients represent differences in transformed GC layer thickness values compared with participants without insulin resistance;unadjusted; adjusted for age, sex, and BMI (model 1); model 1 additionally adjusted for presence of diabetes, presence of systemic hypertension (model 2); model 2 additionally adjusted for IOP (model 3);BMI, body mass index; CI, Confidence Interval; FDR-p, false discovery rate adjusted p; HOMA-IR, homeostatic model assessment of insulin resistance; GCL, ganglion cell layer; Sup, superior; Inf, inferior; Temp, temporal. Insulin resistance was defined as HOMA-IR ≥ 2.34

Table S5. Senstivity analysis for association between hyperinsulinaemia and retinal nerve fibre layer thickness by excluding glaucoma patients

	Unadjusted		Model 1		Model 2		Model 3	
	β (95% CI)	FDR-p	β (95% CI)	FDR-p	β (95% CI)	FDR-p	β (95% CI)	FDR-p
**RNFL Average**	-0.055 (-0.126, 0.016)	0.236	-0.067 (-0.140, 0.005)	0.111	-0.059 (-0.133, 0.015)	0.153	-0.054 (-0.127, 0.020)	0.204
**RNFL Mininum**	-0.094 (-0.171, -0.017)	0.130	-0.112 (-0.194, -0.030)	0.061	-0.109 (-0.192, -0.026)	0.082	-0.110 (-0.194, -0.026)	0.082
**RNFL Temp-sup**	-0.053 (-0.125, 0.019)	0.236	-0.081 (-0.156, -0.006)	0.093	-0.074 (-0.151, 0.002)	0.149	-0.068 (-0.143, 0.007)	0.204
**RNFL Sup**	-0.035 (-0.108, 0.037)	0.388	-0.026 (-0.101, 0.048)	0.488	-0.017 (-0.092, 0.059)	0.665	-0.014 (-0.090, 0.063)	0.724
**RNFL Nasal-sup**	-0.043 (-0.118, 0.032)	0.342	-0.028 (-0.103, 0.047)	0.488	-0.022 (-0.097, 0.053)	0.648	-0.017 (-0.091, 0.057)	0.724
**RNFL Nasal-inf**	-0.065 (-0.136, 0.005)	0.185	-0.063 (-0.134, 0.009)	0.114	-0.059 (-0.132, 0.013)	0.153	-0.058 (-0.131, 0.015)	0.204
**RNFL Inf**	-0.071 (-0.140, -0.001)	0.182	-0.084 (-0.157, -0.010)	0.093	-0.074 (-0.149, 0.000)	0.149	-0.074 (-0.150, 0.001)	0.204
**RNFL Temp-inf**	-0.032 (-0.107, 0.043)	0.401	-0.076 (-0.157, 0.004)	0.111	-0.067 (-0.149, 0.014)	0.153	-0.063 (-0.145, 0.020)	0.204

After excluding 373 participants with glaucoma from the initial cohort of 6,941, the sensitivity analysis was performed on the remaining participants.

For each RNFL thickness parameter, the mean value of the right and left eyes was used in the analysis. RNFL thickness variables were rank-based inverse normal transformed before analysis. β coefficients represent differences in transformed RNFL thickness values compared with participants without hyperinsulinemia.

unadjusted; adjusted for age, sex and BMI (model 1); model 1 additionally adjusted for presence of diabetes, presence of systemic hypertension (model 2); model 2 additionally adjusted for IOP (model 3)

BMI, body mass index; CI, Confidence Interval; FDR-p, false discovery rate adjusted; Sup, superior; Inf, inferior; Temp, temporal; RNFL, retinal nerve fibre layer

Hyperinsulinaemia was defined as values ≥ 13 μIU/mL

Table S6. Senstivity analysis for association between insulin resistance and retinal nerve fibre layer thickness by excluding glaucoma patients

	Unadjusted		Model 1		Model 2		Model 3	
	**β (95% CI)**	**FDR-p**	**β (95% CI)**	**FDR-p**	**β (95% CI)**	**FDR-p**	**β (95% CI)**	**FDR-p**
**RNFL Average**	-0.046 (-0.102, 0.010)	0.145	-0.052 (-0.111, 0.007)	0.166	-0.043 (-0.102, 0.016)	0.304	-0.039 (-0.098, 0.021)	0.363
**RNFL Mininum**	-0.068 (-0.127, -0.009)	0.097	-0.080 (-0.144, -0.016)	0.113	-0.069 (-0.134, -0.004)	0.239	-0.070 (-0.137, -0.003)	0.296
**RNFL Temp-sup**	-0.020 (-0.078, 0.038)	0.570	-0.039 (-0.101, 0.024)	0.262	-0.031 (-0.095, 0.033)	0.389	-0.029 (-0.093, 0.034)	0.416
**RNFL Sup**	-0.060 (-0.119, -0.001)	0.124	-0.043 (-0.106, 0.020)	0.243	-0.036 (-0.098, 0.027)	0.352	-0.035 (-0.099, 0.028)	0.363
**RNFL Nasal-sup**	-0.073 (-0.132, -0.013)	0.097	-0.059 (-0.120, 0.001)	0.166	-0.058 (-0.119, 0.002)	0.239	-0.055 (-0.115, 0.005)	0.296
**RNFL Nasal-inf**	-0.054 (-0.113, 0.004)	0.124	-0.040 (-0.099, 0.019)	0.243	-0.036 (-0.095, 0.023)	0.352	-0.034 (-0.095, 0.026)	0.363
**RNFL Inf**	-0.049 (-0.103, 0.005)	0.124	-0.055 (-0.114, 0.004)	0.166	-0.045 (-0.105, 0.015)	0.304	-0.046 (-0.106, 0.014)	0.353
**RNFL Temp-inf**	0.016 (-0.041, 0.073)	0.574	-0.031 (-0.095, 0.032)	0.332	-0.016 (-0.081, 0.049)	0.625	-0.014 (-0.081, 0.053)	0.680

After excluding 373 participants with glaucoma from the initial cohort of 6,941, the sensitivity analysis was performed on the remaining participants.

For each RNFL thickness parameter, the mean value of the right and left eyes was used in the analysis. RNFL thickness variables were rank-based inverse normal transformed before analysis. β coefficients represent differences in transformed RNFL thickness values compared with participants without insulin resistance.

unadjusted; adjusted for age, sex and BMI (model 1); model 1 additionally adjusted for presence of diabetes, presence of systemic hypertension (model 2); model 2 additionally adjusted for IOP (model 3)

BMI, body mass index; CI, Confidence Interval; FDR-p, false discovery rate adjusted; HOMA-IR, homeostatic model assessment of insulin resistance; Sup, superior; Inf, inferior; Temp, temporal; RNFL, retinal nerve fibre layer

Insulin resistance was defined as HOMA-IR ≥ 2.34

Table S7. Association between hyperinsulinaemia and GCL thickness using untransformed regression analyses

	Unadjusted		Model 1		Model 2		Model 3	
	β (95% CI)	FDR-p	β (95% CI)	FDR-p	β (95% CI)	FDR-p	β (95% CI)	FDR-p
**GCL Average**	-1.145 (-1.840, -0.450)	0.005	-1.087 (-1.757, -0.418)	0.006	-0.925 (-1.602, -0.248)	0.030	-0.917 (-1.596, -0.237)	0.032
**GCL Minimum**	-1.545 (-2.543, -0.547)	0.005	-1.224 (-2.164, -0.284)	0.013	-0.945 (-1.898, 0.008)	0.059	-0.926 (-1.889, 0.038)	0.068
**GCL Temp-sup**	-0.975 (-1.700, -0.250)	0.010	-1.072 (-1.791, -0.352)	0.008	-0.919 (-1.648, -0.190)	0.035	-0.948 (-1.688, -0.209)	0.032
**GCL Sup**	-0.981 (-1.799, -0.163)	0.019	-0.891 (-1.696, -0.086)	0.030	-0.749 (-1.571, 0.073)	0.074	-0.718 (-1.543, 0.107)	0.088
**GCL Nasal-sup**	-1.089 (-1.861, -0.317)	0.008	-1.009 (-1.791, -0.227)	0.013	-0.888 (-1.681, -0.096)	0.044	-0.859 (-1.646, -0.072)	0.050
**GCL Nasal-inf**	-1.476 (-2.242, -0.711)	0.001	-1.301 (-2.065, -0.536)	0.006	-1.124 (-1.895, -0.353)	0.030	-1.115 (-1.887, -0.344)	0.032
**GCL Inf**	-1.260 (-2.053, -0.466)	0.005	-1.075 (-1.875, -0.276)	0.013	-0.881 (-1.690, -0.072)	0.044	-0.872 (-1.695, -0.049)	0.050
**GCL Temp-inf**	-1.028 (-1.765, -0.292)	0.008	-1.106 (-1.854, -0.358)	0.008	-0.920 (-1.680, -0.161)	0.035	-0.914 (-1.681, -0.147)	0.039

For each GC layer thickness parameter, the mean value of the right and left eyes was used in the analysis. β coefficients represent differences in GC layer thickness values compared with participants without hyperinsulinaemia;unadjusted; adjusted for age, sex and BMI (model 1); model 1 additionally adjusted for presence of diabetes, presence of systemic hypertension (model 2); model 2 additionally adjusted for IOP (model 3); BMI, body mass index; CI, Confidence Interval; FDR-p, false discovery rate adjusted p; GCL, Ganglion Cell Layer; Sup, superior; Inf, inferior; Temp, temporal;Hyperinsulinaemia was defined as values ≥ 13 μIU/mL

Table S8. Association between insulin resistance and GCL thickness using untransformed regression analyses

	Unadjusted		Model 1		Model 2		Model 3	
	β (95% CI)	FDR-p	β (95% CI)	FDR-p	β (95% CI)	FDR-p	β (95% CI)	FDR-p
**GCL Average**	-1.023 (-1.592, -0.453)	<0.001	-0.762 (-1.341, -0.183)	0.027	-0.541 (-1.121, 0.040)	0.118	-0.587 (-1.164, -0.010)	0.086
**GCL Minimum**	-1.738 (-2.576, -0.900)	<0.001	-1.238 (-2.054, -0.422)	0.024	-0.824 (-1.637, -0.012)	0.118	-0.886 (-1.702, -0.070)	0.086
**GCL Temp-sup**	-0.808 (-1.397, -0.219)	0.007	-0.756 (-1.387, -0.124)	0.027	-0.551 (-1.176, 0.075)	0.118	-0.613 (-1.237, 0.010)	0.086
**GCL Sup**	-0.962 (-1.620, -0.304)	0.006	-0.633 (-1.305, 0.038)	0.065	-0.397 (-1.070, 0.277)	0.247	-0.430 (-1.101, 0.241)	0.208
**GCL Nasal-sup**	-1.039 (-1.658, -0.419)	0.002	-0.741 (-1.386, -0.096)	0.028	-0.564 (-1.214, 0.085)	0.118	-0.577 (-1.221, 0.068)	0.091
**GCL Nasal-inf**	-1.317 (-1.950, -0.684)	<0.001	-0.887 (-1.530, -0.244)	0.027	-0.666 (-1.321, -0.010)	0.118	-0.700 (-1.359, -0.042)	0.086
**GCL Inf**	-1.186 (-1.840, -0.532)	<0.001	-0.795 (-1.467, -0.123)	0.027	-0.546 (-1.220, 0.127)	0.127	-0.621 (-1.300, 0.058)	0.091
**GCL Temp-inf**	-0.831 (-1.411, -0.251)	0.006	-0.768 (-1.380, -0.157)	0.027	-0.536 (-1.148, 0.076)	0.118	-0.601 (-1.211, 0.008)	0.086

For each GC layer thickness parameter, the mean value of the right and left eyes was used in the analysis. β coefficients represent differences in GC layer thickness values compared with participants without insulin resistance;unadjusted; adjusted for age, sex, and BMI (model 1); model 1 additionally adjusted for presence of diabetes, presence of systemic hypertension (model 2); model 2 additionally adjusted for IOP (model 3);BMI, body mass index; CI, Confidence Interval; FDR-p, false discovery rate adjusted p; HOMA-IR, homeostatic model assessment of insulin resistance; GCL, ganglion cell layer; Sup, superior; Inf, inferior; Temp, temporal. Insulin resistance was defined as HOMA-IR ≥ 2.34
